# Muslim women’s views and experiences of family planning in Saudi Arabia: a qualitative study

**DOI:** 10.1186/s12905-023-02786-2

**Published:** 2023-11-25

**Authors:** Noura Alomair, Samah Alageel, Nathan Davies, Julia V. Bailey

**Affiliations:** 1 Community Health Sciences Department, College of Applied Medical Sciences, King Saud University, Riyadh, Kingdom of Saudi Arabia; 2 Research Department of Primary Care and Population Health, University College London, London, UK

**Keywords:** Muslim women, Islam, Contraception, Family planning, Attitudes, Qualitative study

## Abstract

**Background:**

Islamic beliefs are associated with decreased contraceptive use compared to other religions, and Muslim women’s contraceptive needs are often unmet. Research is needed to provide an in-depth understanding of the complex set of barriers to Muslim women’s contraceptive use. Therefore, we aimed to explore Muslim women’s awareness and experiences with family planning in Saudi Arabia and investigate barriers to contraceptive use and access to family planning.

**Methods:**

A qualitative study using semi-structured interviews with women in a public hospital, in Riyadh, Saudi Arabia, between January and June 2019. Data was analysed using reflexive thematic analysis.

**Results:**

Twenty-eight women participated in the study. Women’s contraceptive awareness was limited to two methods. Women expressed positive attitudes towards family planning and did not believe it is forbidden in Islam. Barriers to contraception use included lack of knowledge, misconceptions, fear of side effects, family and community and social norms. Many women expressed that they have the right to use contraception, even if their husbands disapprove. Our findings show that healthcare providers rarely offered contraceptive advice, even when requested. Healthcare providers often prescribe oral contraceptives without offering information on other methods available.

**Conclusion:**

Our findings suggest that education plays a fundamental role in reproductive autonomy. Men’s role in family planning should be encouraged through sharing reproductive responsibility and supporting women’s contraceptive choices. Efforts should be directed towards improving women’s awareness of different methods of contraception.

**Supplementary Information:**

The online version contains supplementary material available at 10.1186/s12905-023-02786-2.

## Introduction

Access to family planning and contraceptive services and information is a fundamental human right. Women worldwide have the right to be given all resources for well-informed fertility choices [1]. Access to family planning services and contraceptive care improves women’s overall health by reducing unintended pregnancies, unplanned births, miscarriages, and maternal deaths [1]. Vulnerable groups, such as unmarried Muslim women, lack contraceptive care which could have a substantial impact on their overall health, wellbeing, and lives [1]. According to the World Health Organisation, the contraceptive needs of over 200 million women of reproductive age are unmet due to limited access to family planning [1]. Low socio-economic status and low educational attainment are linked with low contraception use [2].

There are several factors affecting unmet needs for family planning among religious communities [1, 3]. Research on the factors influencing Muslim women’s sexual and reproductive health revealed that Muslim women’s contraceptive use is affected by a complex and overlapping set of barriers including personal, family, community, cultural, and religious barriers [3, 4]. Islamic beliefs are associated with decreased contraceptive use compared to other religions [4]. This suggests a complex influence of religious beliefs on contraception access and use. Social and cultural factors might also contribute to this complexity [5, 6].

Women’s reproductive decision-making is influenced by social expectations and gender roles [3]. In low- and middle-income countries women are socially expected to comply with their husbands’ and family members’ reproductive decisions and desires, and in many cases, men are the decision-makers for reproduction [3, 4].

Personal factors also have been associated with access to contraceptive services. Women’s socioeconomic status and educational level have an impact on unmet contraceptive needs [3]. Women’s lack of knowledge of contraceptive methods and fear of side effects act as barriers to contraceptive use [3, 4]. Women’s employment and economic independence are associated with increased reproductive autonomy [4].

In Saudi Arabia, the prevalence of contraceptive use among married women aged 15–49 was 30% in 2018 [7], which is lower than other countries in the Middle East and North Africa region [8]. Several factors influence unmet contraceptive needs in Saudi Arabia. Previous studies highlighted women’s lack of knowledge of different contraceptive methods, misconceptions, fear of side effects, and accessibility issues as the most significant barriers to contraception among Saudi women [9–12].

Although several studies have examined family planning and contraception use in Saudi Arabia, these studies utilised quantitative research methods. Previous studies focused mainly on measuring contraceptive knowledge and reporting on the prevalence of use. Due to the sensitivity and complexity of contraception use and family planning in Muslim communities, using qualitative methodology provides a deeper understanding of Muslim women’s views and experiences. We aimed to explore Muslim women’s awareness, experiences, and attitudes towards family planning and barriers to contraceptive use and access to services in Saudi Arabia.

## Methods

### Design

Qualitative study using face-to-face semi-structured interviews. We chose qualitative research as it can provide valuable and unique understandings that cannot be addressed using quantitative methods. It can provide answers on how quantitative data are created through social processes [13]. Qualitative research aims to provide ‘an in-depth and interpreted understanding of the social world of research participants’ [14].

Data in this paper were collected as part of a larger research project, exploring multiple aspects of sexual and reproductive health among Muslim women in Saudi Arabia [15, 16]. This study was approved by the UCL ethics committee (Reference no. 10157/001) and the hosting hospital, King Fahad Medical City (KFMC) in Riyadh, Saudi Arabia (Reference no. FWA00018774). Interviews took place between January and June 2019.

### Sampling and recruitment

Purposive sampling was used to recruit women aged 18–50. We aimed to recruit women with different educational levels, employment, and marital status. The recruitment process was done by the primary investigator (NA), a young Saudi female public health researcher. It took place in a public hospital, King Fahad Medical City (KFMC), in Riyadh, (the capital city of Saudi Arabia) between January and June 2019. This hospital is one of the major public hospitals in Riyadh that provides primary, secondary, and tertiary healthcare services to the public free of charge. The hospital allowed access to a diverse sample of women and provided a wide variety of insights and experiences.

Potential participants were approached in the women’s health outpatient clinic. Clinic staff assisted in the recruitment process by asking women if they would be interested in taking part in the study, explaining the research, and handing out information sheets. The recruitment process continued until no further themes were emerging and thematic saturation was reached [17].

### Data collection

Semi-structured interviews were conducted face-to-face in a private room in the women’s health outpatient clinic and lasted between 30 to 90 minutes. Interviews were conducted by NA guided by a semi-structured topic guide. The interviewer had no prior relationship with the study participants. Questions were based on the conceptual framework used in Alomair et al. [3], a systematic review exploring factors influencing Muslim women’s sexual and reproductive health. The framework was used to gain a comprehensive understanding of the complex factors influencing contraceptive use. The topic guide included questions on awareness of different contraceptive methods, sources of family planning information, attitudes, and experiences with family planning. Questions also explored perceived barriers to contraception use. Participants were asked about any personal, religious, socio-cultural, and structural factors influencing access and use of family planning. The interview topic guide can be found in Additional file 1.

The topic guide was piloted, and questions were rearranged and rephrased based on the pilot interviews. The piloted interviews were not included in the analysis.

Participants included both married and unmarried women, and in Muslim culture, it is considered unacceptable to assume that unmarried women are using or have used contraception. Nonetheless, it is important to include the accounts of unmarried women as their sexual and reproductive health views and experiences are often underrepresented. All participants were asked the same questions with slight modifications based on marital status to be culturally sensitive and allow participants to answer questions comfortably.

Before the start of each interview, the primary investigator introduced themselves and explained the purpose of the research. Written consent was obtained from participants. Permission to record the interview was also obtained from all participants.

### Data analysis

The data were analysed using reflexive thematic analysis [18]. All audio recordings were transcribed verbatim. All transcripts were checked by NA against the recordings to ensure the accuracy of the transcripts. Interviews were conducted in Arabic and a sample was translated into English for non-Arabic-speaking researchers to contribute to the analysis. All relevant quotes were translated into English for reporting. Data were uploaded to ATLAS.ti for data management and coding. All interviews were initially read by the primary investigator (NA) for data familiarisation. Transcripts were coded by one researcher (NA) and a sample of interviews was double coded by a second researcher (SA). An inductive approach to coding was applied where codes were generated from the data using coding and refinement of themes. All coding and definitions of codes were checked by all authors. Codes were grouped to create the analytical framework and the themes were produced through discussions among all researchers.

We ensured rigour throughout the research process by having a reflexive diary and keeping field notes, providing a clear description of procedures used, offering evidence from the data for all interpretations made, comprehensive analysis of the whole dataset, analysis of divergent cases and disconfirming data, comparing data between and within cases in the dataset, and comparing findings with other studies [17].

## Results

Twenty-eight women were interviewed. Most women were college educated and employed, and over half of the sample was married (*n* = 16). Study participant’s characteristics are presented in Table 1.

**Table 1:** Key characteristics of study participants

Marital status	N
Married	16
Single	9
Divorced	3
Age	N
20–25	7
26–30	3
31–35	8
36–40	7
41–50	3
Number of children	N
0	14
1–2	1
3–5	12
+ 6	1
Education	N
Bachelor’s degree	26
Diploma	2
Employment	N
Employed	18
Unemployed	5
Student	5

Barriers to family planning fell under different domains from the conceptual framework [3] and included personal, family and community factors, which are all influenced by socio-cultural, religious, and institutional factors.

### Awareness about contraception

Women’s awareness of contraceptive methods was mostly limited to oral contraceptive pills (OCP) and intrauterine devices (IUD). A minority of women mentioned patches, injectable contraceptives, and implants, and only one mentioned emergency contraception.

Significant misconceptions regarding modern contraceptives were evident among women. For example, some women believed that hormonal contraceptives could lead to infertility and others thought that IUDs were a permanent contraceptive method.

*“I thought that once you use hormonal IUDs, that’s it, you will not get pregnant anymore. I thought it was permanent.”* **(P12, Married, 25 years old).**

*“I was against the idea of having children right away. But I heard that hormonal contraceptives are dangerous. I heard that especially if a woman never had any kids and used hormonal contraceptives it would cause infertility and other issues.”* **(P12, Married, 25 years old).**

Sources of information on contraception were mainly friends and family members, specifically mothers, sisters, and cousins. Women trusted them more than healthcare providers as a source of information, as they would be speaking from personal experience. Only a few women preferred going to a healthcare provider for contraceptive advice.

*“****P19:*** *I would usually ask my friends, and the internet as a second option.*

***NA:*** *What about talking to a doctor?*

***P19:*** *Umm I don’t know. I feel like I trust people around me with personal experience with contraception. I feel like I trust and value their opinion more than doctors.”* **(P19, Married, 38 years old).**

When it comes to the discussion of family planning, women expressed openness and ease of discussion among other women.

*“It’s a normal discussion. As a woman, you encounter other females in your circle whether it is your mother, grandmother, sister, aunt, cousin, or friend. They will discuss these things [contraception]. It won’t be hidden, and they won’t be secretive about it. It would be openly discussed, at least among women.”* **(P21, Married, 25 years old).**

### Attitudes towards contraception

All women in the sample expressed positive attitudes towards contraception. Women used statements like *“I strongly agree with the use of contraception”,* and *“it was the greatest invention of mankind”* when they were asked about their views towards contraception. Benefits of family planning include physical and emotional benefits for women, financial benefits, and benefits for the children.

*“Of course, I am 100% with contraceptive use. I mean there should be control over fertility. Regardless of the method, I am with, with contraceptive use. Regardless of how you prevent pregnancy, I am with contraceptive use.”* **(P19, Married, 38 years old).**

Many women mentioned how multiple pregnancies and births can have a negative impact on their health and overall well-being. They were grateful that they had the option of birth control to have a healthier body and mind.

*“Pregnancy takes a toll on your body. Everything is different now. Raising kids nowadays is more challenging. You have to live for yourself and enjoy your life and your body.”* **(P14, Married, 38 years old).**

Women highlighted the importance of using contraception to take care of themselves financially and academically, by providing them with the opportunity to focus on their education and career.

*“I didn’t want to get pregnant immediately after marriage. Because I was still in college, and I wanted to focus on my studies.”* **(P18, Married, 36 years old).**

Limiting the number of children was linked with greater opportunities to provide a better quality of life for them. Women mentioned that fewer children means that they have more time to give to each child, and the energy to take care of them and raise them properly. Fewer children were associated with having the financial means to pay for their education, giving them a proper home and good living conditions, in addition to other life necessities.

*“Why would I burden myself, I already have three children, and that is a blessing from God. I can raise them well and give them my all. I mean this is my opinion, but the fewer children you have, the more you can provide for your kids, and give them a better life”* **(P14, Married, 38 years old).**

Some women were sceptical about modern contraceptive methods, believing that they could fail and lead to pregnancy. Failed contraception was experienced by some women which caused emotional distress and made them lose faith in those methods.

*“I had an IUD, and my doctor assured me that I wouldn’t get pregnant. A month later it turned out that I was pregnant. I almost fainted. I wanted to die. I was jumping off the table trying to miscarry.”* **(P18, Married, 36 years old).**

Some women mentioned the importance of using contraception in the first year of marriage to protect themselves and any future children in case of divorce. They discussed the difficulties of having children of divorce. Some women believed that having children might force them to stay in an unhappy marriage and endure an abusive relationship.

*“I am neither with nor against contraception use. But maybe when it comes to the situation [for women] here in Saudi, maybe I am with the use of contraception after marriage, at least for the first 6 or 7 months. Until they get to know each other more. Because if a woman has a baby in the first year of marriage and isn’t happy, this would cause her to endure things she wouldn’t otherwise endure if she didn’t have a baby with that person.”* **(P11, Divorced, 30 years old).**

While contraception use was generally viewed positively by all women in the sample, one participant was judgemental towards women who delay pregnancy early in the marriage, with the perception that the sole purpose of marriage is procreation.

*“I honestly don’t understand why they would use contraception, why did you get married to begin with? ... I know that there is this idea that you should wait a year before getting pregnant. Honestly it is a strange idea, I don’t understand it.”* **(P27, Single, 22 years old).**

### Barriers to contraception use

Several barriers to contraception were mentioned by women. Lack of knowledge about contraception, misconceptions, fear of side effects, and social norms were among the most significant barriers. Some women, specifically women who got married at a young age, did not use contraception before their first pregnancy because they did not know that there were ways to prevent pregnancies.

*“No, I did not use contraception before my first child, I was ignorant, I was only 17 years old. I wasn’t thinking... I didn’t think, I didn’t think about anything.”* **(P20, Divorced, 40 years old).**

Women from an older generation, and in some instances, still in their thirties but perceive themselves to be from an older generation, viewed having children immediately after marriage as customary and expected.

*“In the past, I mean for our generation, we used to get married at a young age. I got married when I was only 20 years old. So, we did not have this idea that you should live your life and then maybe think about having kids. So, for our generation, it was, to get married, and immediately have kids. That was the norm.”* **(P28, Married, 49 years old).**

Some women did not want to use contraception before the first pregnancy, fearing infertility. While some women did not believe that contraception causes infertility, they wanted to confirm their ability to bear children before using any form of contraception. Others however did believe that contraception causes infertility and did not want to risk using them before having at least one child.

*“* ***P22:*** *I didn’t use contraception before my first child.*

***NA:*** *Why?*

***P22:*** *Because we have this culture that if you use contraception in early marriage, it might cause her harm, and prevent her from getting pregnant. It could also delay pregnancy longer than she expected. So, once I had my first baby, I started using contraception.”* **(P22, Married, 38 years old).**

The reason behind some women’s non-use was due to the negative side effects experienced when using modern contraception. Side effects included excessive bleeding, blood clotting, mood changes, weight gain, depression, headache, and changes in menstrual cycle.

*“I considered an IUD but then got scared because I heard it caused excessive bleeding.”* **(P3, Married, 33 years old).**

#### Healthcare provider barriers

Healthcare providers were sometimes a barrier to women’s contraceptive knowledge, access, and use. Healthcare providers rarely offered women detailed advice regarding contraception (e.g., explaining to women how to use certain contraceptives, their side effects, and what to do if the pill was missed). Women also mentioned that healthcare providers will only offer the pill as a contraceptive option, without offering advice on other available methods. Women often referred to written instructions to get their information after consultations.

*“****P1*** *She [the physician] prescribed me contraceptives.*

***NA:*** *Did she explain to you how to use it?*

***P1:*** *Nothing. She told me to take one pill a day, and that’s it… She did not explain to me what this pill does to my body. What happens if I miss a pill? Nothing. I read the Arabic instructions inside the pills packet. But she didn’t tell me anything. She just prescribed it. Didn’t educate me.”* **(P1, Married, 34 years old).**

#### Family and community barriers

Family members including mothers, sisters, mothers-in-law, and sisters-in-law sometimes acted as a barrier to contraceptive use, especially for young women early in a marriage. Husbands tried to influence women’s decision to have more children, sometimes due to the mother-in-law influencing her son’s opinions, pressuring the wife to have more children.

*“If I asked for my husband’s opinion, he would not be accepting of contraception use. He was refusing contraception before having our first baby. But after that, he accepted it. Except for the IUD, he is totally against it. He said that his mother told him that it could lead to infertility. That’s what his mother told him, it’s from her.”* **(P1, Married, 34 years old).**

The opinions of people in the wider community pressured women to get pregnant even when they were not ready. For example, family members or people in women’s social circles warned them about contraception and delaying pregnancy. The experiences of others influenced attitudes towards contraception, for example, stories of women who had used contraception in the past and could not get pregnant, attributing infertility to the use of contraception.

*“****P26:*** *My husband and his entire family were against me using birth control. I told him my mother had similar views, and he said, your mother is right. So, I told him that that’s none of your business [laugh]. He eventually came to terms with my decision.*

***NA:*** *And why is his family against it?*

***P26:*** *It has a personal side to it. His sister couldn’t have babies, she had to go through IVF. So, they are afraid that this might happen to me. My mom used to say if you use it while you are young and never had babies, you might not be able to in the future. So, he was scared because of his family, but now he is convinced.”* **(P26, Married, 22 years old).**

#### Religious views about contraception

Religion did not seem to play any role in women’s contraceptive use or non-use, despite religious rulings forbidding its use. All women in the sample believed that family planning was not against Islamic beliefs. Many women had never heard that family planning is forbidden in Islam.

*“I have never ever heard any religious advisory opinion forbidding contraceptive use.”* **(P21, Single, 34 years old).**

Many women believed that common sense and the overall benefit to humans is consistent with Islamic views. For this reason, many women did not seem to accept or comply with the forbiddance of family planning.

*“There are many beneficial things, but people will say it is Haram [forbidden]. It is also Haram that you bring kids into this world and not take care of them, or don’t have the means to raise them properly. The situation is different now, you can barely have one or two children. So honestly, I don’t know. I don’t feel that it is Haram, but I honestly don’t know. Life is changing and so are those views.”* **(P20, Divorced, 40 years old).**

Women highlighted that there is no explicit text in the Quran or Sunnah that prohibits the use of family planning. When it comes to contraception, religious views are based on the jurisprudence of religious scholars and their interpretations of what is written.

*“There is no explicit text showing the prohibition of contraception. The prophet Muhammad (PBUH) only encouraged marrying and having children …. I mean our religious reference is the Quran and Sunnah. Religious scholar’s interpretations are personal efforts. This is clearly evident in how scholars used to prohibit many things that are now accepted. So we need to use our common sense here.”* **(P19, Married, 38 years old).**

### Control over fertility

The majority of women said that they have control over their fertility choices. When they were asked who the primary decision maker for contraception use was, many said that they had made most of their fertility decisions.

*“I feel most of the time it’s my decision, not his. He is like ‘If you want to get pregnant it’s okay if you don’t want to, it’s okay too. If you want to tie your tubes, it’s also fine if you want to prevent pregnancy however you want it’s your choice’. He is a person who gives me complete freedom and there are no joint decisions.”* **(P2, Married, 37 years old).**

*“The wife, it’s the wife who’s going to be carrying the burden of getting pregnant and taking care of the kids... They worried about these things [contraception] because some family members faced issues using oral contraceptives … So, the woman is responsible for herself, no one will endure her pain or her suffering … Regardless of how involved the husband is. He will never feel her pain, so she should be the one making that decision based on her abilities.”* **(P5, Married, 43 years old).**

Some women said the decision to use contraception should be a shared decision between the husband and wife but felt that it is ultimately the wife who should decide. It was suggested that having an education gave women the courage to make informed decisions and take charge of their fertility.

***“NA:*** *Who do you think should decide?*

***P6:*** *Me, because he doesn’t experience pregnancy and birth. He doesn’t know what I go through. I look back at my mother and her mother, they didn’t have the knowledge, and because of that they did not have the courage to take the decision to stop or say I am done having children. But nowadays, we are all college educated and have the courage to stand up for ourselves and take the decision [to use contraception] even if we had to use it in secret*” **(P6, Married, 34 years old).**

Women who mentioned that the decision to use contraception should be a shared one were mostly unmarried. Married women who said that the couple should decide, also mentioned that they would use birth control regardless of their husband’s opinion if they needed to. They explained that the physical impact bearing children has on women gives them the right to be the primary decision-makers.

*“It should be a shared decision because couples are life partners. The wife shouldn’t use any contraceptives without her husband’s knowledge, especially if he wants to have children. There should be an open discussion regarding these decisions. The couple are sharing a life, they should be sharing all decisions.”* **(P21, Single, 34 years old).**

A husband’s disapproval did not seem to play a significant role or prevent some women from using contraception. Women whose husbands did not want them to use contraception tried negotiating until they reached an agreement.

*“At first, he used to pressure me and tried to convince me to have a fourth child. But I convinced him. I was like thank God we have two girls and a boy; is there a third kind we don’t have? Is there something else that would make you want another baby? What are we looking for exactly? At first, he was upset with me, and now he just laughs about it. He is now convinced that I am right. We have been married for a long time, and he keeps telling me that ‘your views were right, I’m glad I listened to you’.”* **(P14, Married, 38 years old).**

However, this was not the case for all women, where some women in the sample explained that their husbands were the decision-makers over reproductive choices. Some women were unable to use contraception due to their husband’s refusal. This has led them to hide their contraceptive use from their disapproving husbands. For example, a 44-year-old participant, with seven children, explained that she would like to stop having children, but could not influence her husband’s decision.

***P7:*** *If it was up to me, I would like to tie my tubes and stop having kids, but my husband won’t allow it. After my last two miscarriages, He said you can take a break for three months before the next pregnancy… I’m getting older and already have a special needs kid that takes all my energy… I thought about the injectables because my husband won’t find out, but the doctor said it might cause heavy bleeding, and I said I don’t mind, I just need to stop getting pregnant. He doesn’t like me using any birth control so he can feel the IUD and I don’t want him to find out.*

***NA:*** *Are you planning to use contraception without telling him?*

***P7:*** *Yes. I can’t do this anymore and honesty hasn’t benefited me so far.”* **(P7, Married, 44 years old).**

Overall, married women showed control over fertility decisions. Being armed with knowledge seemed to play a significant role in empowering women to take charge of their own fertility. Even when there were external barriers including husbands, family members, and social networks.

### Responsibility for preventing pregnancy

The burden of preventing pregnancy seemed to fall solely on women. There was a general acceptance of the fact that women are completely responsible for birth control. Some women even said that they did not know that birth control could be used by men.

*“I didn’t know that men could do something about preventing pregnancy until a couple of years ago, it’s not well known, maybe married people talk about it in my absence.”* **(P2, Single, 22 years old).**

A significant part of women having to shoulder the sole responsibility of family planning is the perception that men do not take their desire to control fertility seriously. Many mentioned that men do not care if contraception fails, and in some instances, it was believed to be the husband’s intention. The only way a man would be willing to share the responsibility of family planning was if he had a strong desire to stop having children. If finances were not an issue, men were less inclined to limit the number of children or encourage the use of contraception.

*“I think the wife is more committed [to preventing pregnancy]. Men would be okay if their wives kept getting pregnant… The wife will be more responsible when using contraceptives, she’s the one facing the burden of giving birth and raising children. However, men are okay with having more kids, if his wife had a baby every year, he would be okay with that. The wife is more committed to family planning as she’s responsible for raising children. Men are not faced with any consequences, if the husband is financially able, he would be okay with having many children. I don’t know any couple where the husband is responsible for family planning.”* **(P1, Married, 34 years old).**

The side effects of hormonal contraceptives were bothersome for some women. Although it was perceived to be easier for men to use contraception (i.e., condoms, withdrawal), it was uncommon in their culture for men to share responsibility for preventing pregnancy. It is both expected and accepted for women to bear contraceptive responsibility alone.

*“****NA:*** *Was your husband accepting the use of male contraceptive methods? Or you didn’t discuss it with him?*

***P19:*** *No, I didn’t even discuss it with him, I don’t think he will be open to it. I feel like he wouldn’t even accept or comprehend the idea. It’s a bit foreign to him. Even if he tried, let’s say there’s a pill he can take, he wouldn’t be as committed. He wouldn’t accept it, even for something like condoms, for example, they feel that it challenges their masculinity. Because it’s called birth control, the name makes them feel like it’s a woman’s job, because it prevents pregnancy and women get pregnant therefore, they should be responsible for it.”* **(P19, Married, 38 years old).**

Condom use as a birth control method was not identified by many women and was perceived to be insulting men’s masculinity. It was also generally accepted for men to refuse condom use as a birth control option.

*“Some methods, for example condoms, challenge a man’s masculinity. Many men don’t prefer using condoms, and that’s their right.”* **(P8, Single, 22 years old).**

Only one woman mentioned her husband offering to use withdrawal or condoms when she had negative side effects from hormonal contraceptives. Even then, the woman refused her husband having responsibility for family planning.

*“The side effects [of the pill] were really bothering me. I was very anxious, it started to affect my mental health. I really did not want to get pregnant. He told me that we could use natural methods [withdrawal] or condoms. We can arrange something, but I could not trust it. I was scared. There was a discussion because of the side effects I was experiencing but I completely shut down his offer.”* **(P23, Married, 29 years old).**

## Discussion

Women’s awareness of contraception was limited to two types of contraceptive methods (IUD and OCP). All women expressed positive attitudes toward contraception and were supportive of limiting the number of children to provide a better quality of life for themselves and their children. Barriers to contraception use include lack of knowledge about contraception, misconceptions, fear of side effects, healthcare providers’ attitudes, and social norms.

Lack of knowledge about reproduction and family planning is one of the most significant barriers to preventing unwanted pregnancies worldwide [3]. Women who are most vulnerable to lacking contraceptive awareness often receive information and advice only after they have had many, closely spaced, births [19]. Providing women with knowledge can empower them to be decision-makers over their reproductive choices, leading to better health outcomes and life satisfaction. However, in the absence of credible sources of information, women often receive misleading and inaccurate information, causing misconceptions that can hinder effective contraceptive use. Information provision requires an understanding of socio-cultural barriers and common misconceptions to improve women’s overall contraceptive awareness and use.

Saudi Women’s knowledge of contraceptive methods was often limited to the most commonly used methods. Most research on Saudi women’s contraceptive knowledge focuses exclusively on OCP, with high levels of knowledge reported regarding this method [20]. OCPs are the most commonly used method among women in Saudi Arabia, followed by the IUD [20]. Our findings showed that women only received medical advice on OCPs and IUDs, with healthcare providers often prescribing the pill without any advice. This could explain why these are the two most commonly used methods in Saudi Arabia. Evidence from the United Kingdom shows similar findings, where physicians recommended OCPs without providing information on other contraceptive methods [21]. Physicians tend to favour OCPs because they are effective in preventing pregnancy, readily available, and easy to use [22]. Healthcare providers have also been known to deny access to a specific contraceptive method due to their own prejudice regarding the method or its delivery system [22].

Most women in our study were college educated and able to control their fertility choices. Women felt that they have the right to decide when to get pregnant, and how many children they have, as they will be bearing, and often have the sole responsibility of raising and caring for their children. This contradicts existing evidence on Muslim women’s contraceptive decision-making. Traditionally, husbands and older family members tend to have the prime control over women’s fertility choices [3]. Many Saudi women reported acquiring informed consent from their husbands as the main barrier to hormonal contraceptive use [23]. However, most women in our study felt that they should have the right to control their reproduction, and in some instances, believed they had the right to hide their contraceptive use from their husbands if they disapproved. It is important to note that all women in our research were highly educated and said that education had empowered them to take charge of their own fertility. Education’s contributions to women’s empowerment, particularly with regard to reproduction, have been previously highlighted as key to women’s reproductive autonomy [24].

The responsibility of preventing pregnancy falls disproportionately on women in all settings globally [2, 25, 26]. In addition to the physical burden, preventing pregnancies can be emotionally and mentally taxing [25]. Reasons why women take full responsibility include their lack of awareness that there are methods that could be used by men, partners not taking the woman’s desire to prevent pregnancies seriously, condoms being associated with disease and promiscuity, and condoms being viewed as undermining men’s masculinity [26–28]. Viewing pregnancy as something that physically happens to women also shapes men’s belief that birth control is “a woman’s responsibility” [26].

Religion is a significant barrier to Muslim women’s contraception use. Several studies conducted in Saudi Arabia found that many Saudi women believe that it is against Islamic beliefs to use contraception [20, 29]. Yet, our research showed opposing findings, where religion did not seem to act as a barrier to Saudi women’s contraceptive use. A study conducted on Saudi women’s views towards emergency contraception showed similar findings, where women did not believe that emergency contraception is forbidden in Islam [30]. Family planning is a highly debated subject in Islam. Religious scholars supporting the use of family planning stated that there are no clear texts in the Quran and Sunnah that forbid its use [3]. Islam encourages high fertility, and having many children is something that many Muslims adopt in order to become good Muslims [3]. Some scholars have interpreted an Islamic emphasis on high fertility as forbidding family planning. These different views lead to inconsistencies in contraception use among Muslim women, both in Saudi and worldwide, depending on the religious doctrine followed [3].

### Strengths and limitations

An important strength of this study is the diversity of the sample in terms of including the underrepresented accounts of unmarried Muslim women. Previous studies only included married women due to the perceived cultural unacceptability of asking unmarried women about contraception or sexual health [3]. We interviewed women from different age groups from 20 to 50 years old, representing different views and generational experiences with family planning.

The transferability of the study findings to other contexts may be limited due to the specific urban setting. Participants from other cities in Saudi Arabia may have more conservative views regarding contraception.

Interviews may have been influenced by the power imbalance between the primary investigator and participants which could lead to social desirability bias. However, the primary investigator is a young Saudi woman, which helped establish a stronger rapport, and women were more comfortable sharing personal views. Additionally, the primary investigator focused on adopting an approach of non-judgemental curiosity during interviews. The research team are all advocates for women’s health which might have influenced the research aims and informed the interpretation of the data. The authors attempted to be reflexive throughout all stages of this project, being aware that our interests and beliefs may have influenced the research process.

### Research and policy implications

There is no evidence of contraceptive counselling and healthcare providers’ contributions to Saudi women’s contraceptive use [20]. Further research is needed to explore Muslim women’s experiences with contraceptive counselling. All studies that examined Saudi women’s contraceptive use recommend effective family planning counselling and education to improve women’s awareness of all available contraceptive methods. Effective counselling increases women’s awareness of the different choices they have, therefore improving informed choice, increasing contraceptive use, and most importantly effective use.

Efforts working towards normalising men’s contribution to fertility regulation are highly needed. It has the potential to improve men’s engagement, thus relieving women from having to bear the sole physical and emotional burden of family planning. Future research should focus on exploring men’s views towards sharing the responsibility for fertility regulation and exploring the possible barriers to men’s contraceptive use.

## Conclusion

This study suggested that educated Saudi women exhibited control over their fertility choices and believed they had the right to decide when, and how many children they have. Education plays a fundamental role in reproductive autonomy. Efforts should be directed at improving women’s awareness of different methods of contraception. Men’s role in family planning should be encouraged through sharing reproductive responsibility and supporting women’s contraceptive choices.

### Supplementary Information

**Additional file 1. **

## Data Availability

Data are not publicly available due to concerns of participant confidentiality. Requests for access to the underlying data may be directed to the UCL Research Ethics Committee: ethics@ucl.ac.uk.
