# Supplementary material for: Muslim women’s views and experiences of family planning in Saudi Arabia: a qualitative study
Source: BMC Womens Health. 2023 Nov 25;23:625. doi: 10.1186/s12905-023-02786-2 (PMC10675866; doi:10.1186/s12905-023-02786-2)
Supplement: Supplementary file 1 — Additional file 1. [file 12905_2023_2786_MOESM1_ESM.doc]

**Topic guide**

1. **Introduction**

- Thank you for taking part in the study.
- Introduction to researcher.
- Explain to the participants why they have been chosen to take part and explain the aims and objectives of the study.
- Explain confidentiality and anonymity, reassure participants that all answers are confidential, and data will be anonymised.
- Reconfirm that they are happy to be recorded, explain length of interview and nature of the discussion.
- Remind participants that they may stop at any time and that they don’t need to answer any questions they wouldn’t feel comfortable answering.
- Sign the consent form.
- Check if they have any further questions and if they are happy to continue.

1. **Socioeconomic and family characteristics**

Demographic sheet (ID, age, level of education)

**For this study, we are interested in women’s health in terms of relationships, pregnancy and contraception.**

**So, when you hear the term sexual and reproductive health, what do you think this means or involve?**

Where have you learned about sexual and reproductive health issues?

- Probe: School/home/parents/youth group /friends /media /Internet.
- How was the teaching delivered? (Video, class, written material)

What did you learn about?

How did you find this information?

- Probe: Useful, informative vs. basic, useless – why or why not?

What would you like to have been taught about/known more about?

1. **Relationship status**

Are you in a relationship at the moment?

Married or planning to be married?

1. **Contraceptive methods knowledge/ attitudes**

Could you tell me what you know about birth control methods?

- Probe (mode of action, types)

Do you know what contraception is available in Saudi?

- What types are available for men? and women?

How and where did you learn about those methods?

Do you know the place or person where you could obtain contraception?

Did you [or] do you plan on consulting a doctor before using contraception?

- Why/why not

What does your religion say about family planning?

- Do you think is halal to use contraceptives?

What does your family/community say about using contraceptives?

- What do you think about their views?
- Does their view influence your use?
- Do you think community’s views would influence other women’s use?

[If relevant] What does your partner say about using contraceptives?

- Do you have similar views regarding family planning?

Who do you think should be involved in deciding what methods to use?

- You, partner or both?

Is it mainly a woman’s responsibility to ensure that contraception is used regularly?

- Why/why not?

What would prevent you from using contraception?

Are you worried about using contraception?

[If okay to ask] What is your experience with using contraception?
